# The Role of Chloride Incorporation in Lead‐Free 2D Perovskite (BA)_2_SnI_4_: Morphology, Photoluminescence, Phase Transition, and Charge Transport

**DOI:** 10.1002/advs.201802019

**Published:** 2019-01-20

**Authors:** Jun Wang, Hongzhi Shen, Wancai Li, Shuai Wang, Junze Li, Dehui Li

**Affiliations:** ^1^ School of Optical and Electronic Information Huazhong University of Science and Technology Wuhan 430074 China; ^2^ School of Optical and Electronic Information and Wuhan National Laboratory for Optoelectronics Huazhong University of Science and Technology Wuhan 430074 China

**Keywords:** charge transport, chloride incorporation, morphology, phase transitions, photoluminescence

## Abstract

The incorporation of chloride (Cl) into methylammonium lead iodide (MAPbI_3_) perovskites has attracted much attention because of the significantly improved performance of the MAPbI_3_‐based optoelectronic devices with a negligible small amount of Cl incorporation. It is expected that the Cl incorporation in 2D perovskites with layered nature would be much more efficient and thus can greatly alter the morphology, optical properties, phase transition, and charge transport; however, studies on those aspects in 2D perovskites remain elusive up to date. Here, a one‐pot solution method to synthesize the Cl‐doped lead‐free 2D perovskite (BA)_2_SnI_4_ with various Cl incorporation concentrations is reported and how the Cl incorporation affects the morphology change, photoluminescence, phase transition, and charge transport is investigated. The Cl element is successfully incorporated into the crystal lattice in the solution‐processed perovskite materials, confirmed by X‐ray photoelectron spectroscopy and energy dispersive X‐ray spectroscopy measurements. The temperature‐dependent photoluminescence studies indicate that the emission properties and phase transition behavior in (BA)_2_SnI_4−_ *_x_*Cl*_x_* can be tuned by varying the Cl incorporation concentration. Electrical measurement suggests that the charge transport behavior can also be greatly altered by the Cl doping concentration and the electrical conductivity can be significantly improved under a higher Cl incorporation concentration.

Three‐dimensional (3D) organic–inorganic halide perovskites such as methylammonium lead iodide (MAPbI_3_) have been extensively studied in past few years due to their ease of fabrication, low cost, and high‐power conversion efficiency,^1^, ^2^, ^3^, ^4^ thus holding the promise for the cost‐effective solar cells and other diverse optoelectronic applications such as lasers,^5^, ^6^ photodetectors,^7^, ^8^ and light‐emitting devices with decent performance.^9^, ^10^, ^11^ The certified power conversion efficiency of the perovskite‐based solar cells has rapidly soared up to 23% within several years.^4^ Despite the rapid advancement of the optoelectronic applications based on 3D perovskites, their inherent long‐term environmental instability and the toxicity of lead severely limit them to be widely applied in industry.^12^, ^13^, ^14^

To address this long‐term instability issue, two‐dimensional (2D) organic−inorganic layered perovskites based on the lead halide framework with great environmental stability are emerging as a new class of materials and thus have attracted significant attention recently.^15^, ^16^ 2D perovskites have the general chemical formula of (A)_2_(B)*_n_*_−1_M*_n_*X_3_ *_n_*_+1_ where A, B are cations, M is a divalent metal, X is a halogen, and the integer *n* represents the layer number of inorganic layers sandwiched between two organic layers.^17^, ^18^, ^19^ In addition to the environmental stability, 2D perovskites are equipped with tunable bandgap energy in the entire visible range by changing the layer number *n* and/or chemical compositions, and large exciton binding energy arising from dielectric confinement and quantum confinement effect, which provide an interesting system to explore for the novel optoelectronic devices.^20^, ^21^ Furthermore, the layered nature of 2D perovskites allows us to integrate them with other 2D layered materials to extend their functions as demanding.

Except for the long‐term environmental stability,^22^ the toxicity of lead casts a shadow on the large‐scale perovskite‐based optoelectronic applications in practice in both 2D and 3D perovskite materials.^23^ There has been a growing effort to replace lead perovskites with environment‐friendly lead‐free compounds by substituting the divalent lead (Pb) with nontoxic metals including bismuth (Bi), germanium (Ge), and tin (Sn).^24^, ^25^, ^26^, ^27^ Unfortunately, due to the inherent propensity for the oxidation of Sn^2+^ to Sn^4+^, which creates a high defect density and hence lability, 3D Sn‐based perovskite materials undergo rapid degradation and thus show poor reproducibility even prepared in a glovebox with trace amounts of water and oxygen.^28^ It is expected that 2D perovskites with layered nature can efficiently prevent the inorganic layer from being directly contacted with water in ambient conditions, resulting in the greatly improved environmental stability of Sn‐based perovskites.^29^, ^30^, ^31^ Nevertheless, studies on 2D Sn‐based perovskites still largely remain unexplored.

It has been demonstrated that chlorine incorporation in the MAPbI_3_ can dramatically improve the performance of MAPbI_3_ based optoelectronic devices even with trace amounts of Cl doping.^32^, ^33^ This is largely because the Cl incorporation can alter the crystallization process and thus improve the morphology, thickness, coverage, and uniformity of the as‐synthesized perovskite films.^34^ As a consequence, the carriers’ diffusion length is greatly increased (from ≈100 nm to over 1 µm) and the electron–hole recombination rate is significantly suppressed, leading to the improved device performance.^35^, ^36^ Nonetheless, previous studies theoretically predicate that the Cl incorporation concentration is less than 3–4% in MAPbI_3_ and no Cl—Pb bonds are experimentally detected, suggesting that the Cl element is not really incorporated into the crystal lattice of MAPbI_3_.^37^ The theoretical calculations also reveal that Cl atoms prefer to occupy the apical positions of [PbI_6_]^4−^ octahedrons in MAPbI_3_ and thus it is expected that Cl incorporation concentration can reach a much higher value in 2D perovskites due to their layered nature. This has been experimentally demonstrated in 2D perovskites that a high Cl incorporation ratio can be achieved by using vapor phase growth method.^38^ Similarly, we anticipate that Cl incorporation can also be achieved in the solution‐processing 2D perovskite materials. To this end, it is of great significance to synthesize nontoxic Sn‐based 2D perovskites with various Cl incorporation concentration and investigate their optical and charge transport properties for potential optoelectronic applications.

Herein, we for the first time report on a one‐pot method to synthesize the lead‐free (BA)_2_SnI*_x_*Cl_4−_ *_x_* (BA = *n*‐CH_3_CH_2_CH_2_CH_2_NH_3_)2D perovskites and investigate how the Cl incorporation concentration influences the morphology, phase transition, optical and charge transport properties in order to optimize device performance. The morphology of the resultant samples changes from plates to needles with the increase of the Cl incorporation concentration. X‐ray photoelectron spectroscopy (XPS) and energy‐dispersive X‐ray spectroscopy (EDS) reveal the presence of Cl element and the formation of the Sn—Cl bonds in our samples, which has not been observed in solution‐processing 3D perovskites up to date. The temperature‐dependent photoluminescence (PL) and differential scanning calorimeter (DSC) studies suggest that the Cl doping concentration can significantly change structural phase transition temperature. Finally, with the increase of the Cl doping ratio, the electrical conductivity is greatly enhanced and the charge transport changes to variable‐range hopping, indicating that Cl doping introduces the extra crystal structure disorders.

(BA)_2_SnI_4_ crystals with different Cl incorporation concentration were synthesized by using the method similar to previous report except that HI solution was replaced by an excess of aqueous HCl/H_3_PO_2_ solution in order to incorporate the Cl element into the (BA)_2_SnI_4_ crystals.^39^ Previous studies show that the strong reducing properties of H_3_PO_2_ can not only prevent the oxidation of Sn^2+^ to Sn^4+^ but also suppress the oxidation of I^−^ to I_3_^−^ and/or I_2_. Thus, we properly increase the amount of the H_3_PO_2_ solution so as to obtain the (BA)_2_SnI_4−_ *_x_*Cl*_x_* without Sn^4+^. The ratio of I:Cl in these solutions was gradually increased from 1:0 to 1:8 by adding different amount of BAI solution. It should be noted that the I:Cl ratio stands for the volume ratio of BAI in 57% w/w aqueous HI solution to 37% w/w aqueous HCl solution.

**Figure** **1**a shows the possible crystal structures of (BA)_2_SnI_4_ with the Cl incorporation. As previously reported, the Cl atoms incorporated into the crystal lattice prefer to occupy the apical positions of [PbI_6_]^4−^ octahedrons but still have a possibility to occupy the equatorial positions of [PbI_6_]^4−^ octahedrons as well.^40^ The exact positions the Cl occupied can be extracted from single‐crystal X‐ray diffraction (XRD) measurement but are still unrevealed due to the polycrystalline nature of our synthesized samples and thus we indicate the possible positions with dashed lines in Figure 1a. The morphology of the as‐synthesized (BA)_2_SnI_4−_ *_x_*Cl*_x_* with different ratio of I:Cl is shown in Figure 1b. The shape of the as‐synthesized crystals changes from the thin flake with a size of ≈cm without Cl and gradually to short and thin needle‐like shape when the ratio of I:Cl increases to 1:8. The presence of Cl ions would lead to the formation of new nucleation centers that assist the growth of the perovskite crystals, thus altering the nucleation and growth process compared to that without Cl incorporation.^41^ As a result, the shape of the resultant (BA)_2_SnI_4−_ *_x_*Cl*_x_* evolves from flake‐like to needle‐like with the increase of the Cl concentration in the precursor solution. In addition to the shape evolution, the surface morphology of the resultant (BA)_2_SnI_4−_ *_x_*Cl*_x_* can also be tuned with the Cl concentration. In comparison with the rough surface of the (BA)_2_SnI_4_ crystals (Figure 1c), (BA)_2_SnI_4−_ *_x_*Cl*_x_* shows a much smooth surface (Figure 1d,e), similar to 3D perovskite case when the Cl incorporation can be applied to control the morphology and uniformity of the synthesized 3D perovskite films.^34^, ^36^ Scanning electron microscopy (SEM) image of the edge of (BA)_2_SnI_4−_ *_x_*Cl*_x_* needles clearly indicates that (BA)_2_SnI_4−_ *_x_*Cl*_x_* needles maintain the layered nature after Cl incorporation (Figure 1e), which is further confirmed by XRD patterns discussed below.

**Figure 1:**
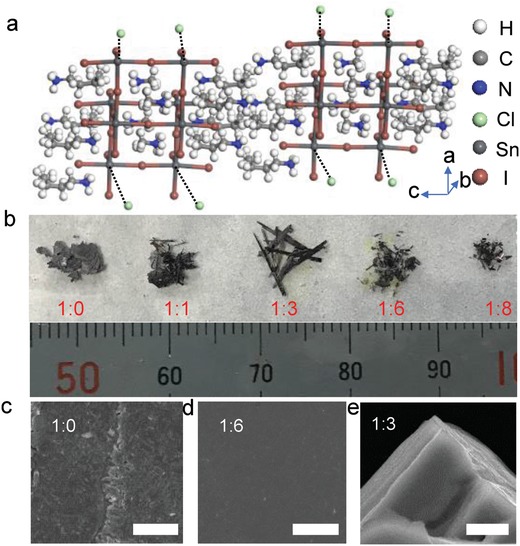
Typical morphology for the as‐grown (BA)_2_SnI_4−_ *_x_*Cl*_x_* crystals: a) The possible crystal structures of (BA)_2_SnI_4−_ *_x_*Cl*_x_*. b) Photograph of the as‐synthesized crystals with various Cl incorporation concentration. c) Top‐view SEM image of (BA)_2_SnI_4_ shows a rather rough surface. The scale bar is 50 µm. d) Top‐view SEM image of the as‐synthesized crystals with the I:Cl ratio of 1:6. The scale bar is 50 µm. e) SEM image of the edge of (BA)_2_SnI_4−_ *_x_*Cl*_x_* needles clearly indicates that (BA)_2_SnI_4−_ *_x_*Cl*_x_* needles maintain the layered nature. The scale bar is 5 µm.

Previous studies have theoretically revealed that the Cl incorporation concentration cannot surpass 3–4% due to the very large ionic radii difference between Cl^−^ and I^−^ anions.^42^ In the experimental aspect, it is widely reported that the XRD patterns of the Cl doped MAPbI_3−_ *_x_*Cl*_x_* films have the same diffraction peaks as that of pure MAPbI_3_ films and no Cl—Pb bonds has been observed from XPS spectrum of MAPbI_3−_ *_x_*Cl*_x_* films, both of which suggest that the Cl element is not truly incorporated into the crystal lattice of MAPbI_3_.^43^ Nevertheless, we found that the diffraction peaks in our (BA)_2_SnI_4−_ *_x_*Cl*_x_* crystals gradually shift to the smaller angles as the ratio of I to Cl in precursor increases, which agrees with previous reports and suggests^44^ that the Cl element has been successfully doped into the crystal lattice of (BA)_2_SnI_4_ (**Figure** **2**a and Figure S1, Supporting Information). The Cl incorporation leads to the lattice distortion, resulting in the shift of the diffraction peaks. The presence of diffraction peaks below 10° for all samples shows that the crystals after Cl doping still maintain the 2D layered nature.^45^ Furthermore, the disappearance of the diffraction peaks with the increase of Cl incorporation concentration would be possibly due to the change of the crystal orientation for the samples with different Cl incorporation concentration.

**Figure 2:**
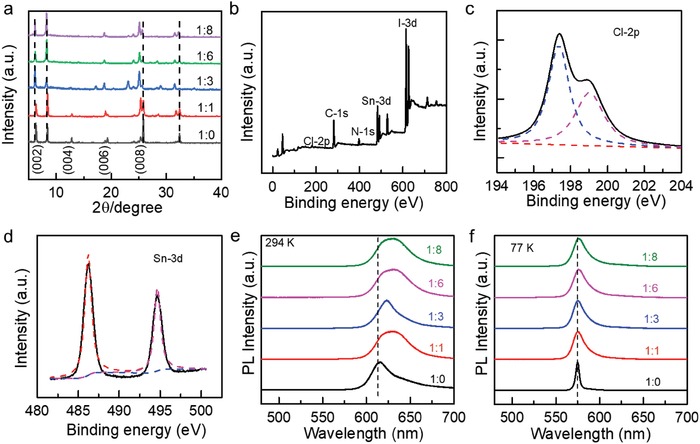
Material characterizations for (BA)_2_SnI_4−_ *_x_*Cl*_x_* crystals: a) XRD patterns of as‐synthesized crystals with various Cl doping concentration. b) XPS spectrum for the (BA)_2_SnI_4−_ *_x_*Cl*_x_* crystals with the I:Cl ratio of 1:3 in the precursor solution. c) Zoom‐in XPS spectrum of Cl 2p electrons indicates Cl element has been incorporated into the crystal lattice of (BA)_2_SnI_4_. d) Zoom‐in XPS spectrum of Sn (3d) peak shows the absence of Sn^4+^. e,f) Steady‐state PL studies at room temperature and 77 K.

To further confirm that the Cl element indeed incorporates into the crystal lattice of (BA)_2_SnI_4_, we have carried out the XPS studies for the as‐synthesized crystals with various Cl doping concentration. Figure 2b displays the XPS spectrum for the (BA)_2_SnI_4−_ *_x_*Cl*_x_* crystals with the I:Cl ratio of 1:3 in the precursor solution, which clearly shows that the Cl 2p electrons are present. Close inspection of Cl (2p) peak indicates that two peaks are present with the binding energies of 197.7 and 199.4 eV (Figure 2c), corresponding to the energy of Cl—Sn bonds, which is another strong evidence that Cl element has been incorporated into the crystal lattice of (BA)_2_SnI_4_.^46^ The presence of Cl element in the resultant (BA)_2_SnI_4−_ *_x_*Cl*_x_* has been further verified by EDS studies, which reveals that the Cl element gradually increases as the ratio of I to Cl in precursor solution increases (Figure S2, Supporting Information). Although the I:Cl ratio measured by EDS is not accurate, the trend of the ratio unambiguously indicates the Cl incorporation ratio increases as the increase of the I:Cl ratio in precursor solution. This is the first time that Cl element is successfully doped into the I‐based organic–inorganic halide perovskites by solution method.

The XPS spectra were also used to identify whether there is Sn^4+^ within our samples due to the easy oxidation of Sn^2+^ to Sn^4+^.^47^ The Sn (3d) peak can be well indexed to Sn^2+^ without the presence of Sn^4+^, which might be due to the excess strong reducing H_3_PO_2_ solution we used during the growth process (Figure 2d). For comparison, we also measured XPS spectrum of pure (BA)_2_SnI_4_ without any Cl doping and no Sn^4+^ peak was observed either (Figure S3, Supporting Information). To conclude, all those evidences point to that our one‐pot solution method by using excess H_3_PO_2_ solution provides an efficient route to synthesize the Cl‐doped Sn^4+^‐free tin‐based 2D perovskites for further optoelectronic applications.

To investigate how the Cl incorporation concentration influences the optical properties of the as‐synthesized (BA)_2_SnI_4−_ *_x_*Cl*_x_* crystals, we have carried out the steady‐state PL studies at room temperature and 77 K. With the increase of the I:Cl ratio, the emission peak continuously shifts from 615 to 630 nm and the full width at half‐maximum (FWHM) simultaneously broadens, which might be due to the Cl incorporation introduced the doping effect, lattice distortion, and disorders.^48^ While the doping effect and/or the lattice distortion induced by Cl incorporation can give rise to the bandgap narrowing effect and thus the redshift of the emission peak, the disorders respond to the peak broadening. At 77 K, only one emission peak was observed, and no defect emission is present for all samples, suggesting the excellent crystalline quality of the as‐grown samples. While the FWHMs were also greatly narrowed at 77 K, the emission peak only slightly shifts from 575 to 576 nm with the increase of the I:Cl ratio. The narrowing of the FWHMs can be ascribed to the reduction of the thermal broadening, whereas the reasons for the reduced peak shift are possibly due to the freezing of the motion of disorders and the phase transition (Figure S4, Supporting Information).^49^ We also measured the reflection spectra for samples with different Cl incorporation ratios, from which the absorption onset can be derived (Figure S5, Supporting Information). The absorption onset from reflection spectra shows a gradual redshift with the increase of the Cl incorporation concentration, consistent with the steady‐state PL studies at room temperature and 77 K (Figure 2e,f).

To fully explore the optical properties of (BA)_2_SnI_4−_ *_x_*Cl*_x_* crystals, temperature‐dependent PL studies were further carried out on the exfoliated (BA)_2_SnI_4−_ *_x_*Cl*_x_* microplates. To minimize the degradation of the exfoliated (BA)_2_SnI_4−_ *_x_*Cl*_x_* microplates, we put the samples immediately into the vacuum chamber once they are prepared. **Figure** **3**a,c displays the temperature‐dependent PL mapping of the (BA)_2_SnI_4_ and (BA)_2_SnI_4−_ *_x_*Cl*_x_* with an I:Cl ratio of 1:8 in precursor solution, respectively, excited by a 473 nm continuous wave (CW) laser. For both samples, the emission peak shows a blueshift with the reducing temperature and a sudden peak shift was observed at a certain temperature depending on the cooling and heating cycle, and the Cl incorporation. If we extracted the emission peak for both the cooling and heating cycle, an apparent hysteresis is observed with a temperature span of 20 K for (BA)_2_SnI_4_ and 40 K for (BA)_2_SnI_4−_ *_x_*Cl*_x_* with an I:Cl ratio of 1:8, respectively, between the cooling and heating cycle (Figure 3b,d). The similar emission peak shift trend and hysteresis loop between the cooling and heating cycle has been observed for (BA)_2_SnI_4−_ *_x_*Cl*_x_* with other different I:Cl ratio (Figure 3e and Figure S6, Supporting Information). This indicates that this sort of behavior is common for all samples.

**Figure 3:**
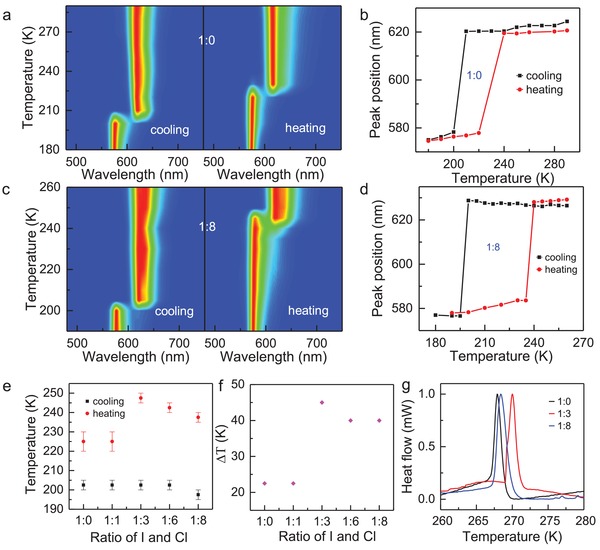
Optical properties and phase transition of (BA)_2_SnI_4−_ *_x_*Cl*_x_* crystals: a) Temperature‐dependent PL mapping of the (BA)_2_SnI_4_ for the cooling and heating cycle. b) The temperature‐dependent emission peak of (BA)_2_SnI_4_ for both the cooling and heating cycle extracted from (a). c) Temperature‐dependent PL mapping of (BA)_2_SnI_4−_ *_x_*Cl*_x_* with an I:Cl ratio of 1:8 in precursor solution. d) The temperature‐dependent emission peak of (BA)_2_SnI_4−_ *_x_*Cl*_x_* with an I:Cl ratio of 1:8 extracted from (c). e) Summarized phase transition temperatures of both the cooling and heating cycle for (BA)_2_SnI_4−_ *_x_*Cl*_x_* with various Cl incorporation concentration. f) The temperature span between the cooling and heating cycle for (BA)_2_SnI_4−_ *_x_*Cl*_x_* with various Cl incorporation concentration. g) DSC plots of the as‐prepared (BA)_2_SnI_4−_ *_x_*Cl*_x_* crystals with a heating rate of 2 °C min^−1^.

Previous studies reveal that the aliphatic materials (C*_n_*H_2_ *_n_*_+1_NH_3_)_2_MX_4_ often exhibit a range of temperature‐dependent structural phase transitions, which are associated with changes in the ordering and hydrogen bonding of the organic cations.^50^, ^51^ How this sort of structural phase changes in (C*_n_*H_2_ *_n_*_+1_NH_3_)_2_SnI_4_ compounds affects their optical properties has been investigated before, based on which we are able to attribute the sudden emission peak shift to the orthorhombic‐to‐orthorhombic phase transition with a phase transition temperature of 254.2 K.^51^ The crystal structure before and after the phase transition all show K_2_NiF_4_ structure type with a cell setting of orthorhombic (space group: *Pbca*). The lattice constants for the low‐temperature phase are *a* = 8.932 Å, *b* = 26.023 Å, *c* = 8.408 Å and for the high‐temperature phase are *a* = 8.814 Å, *b* = 8.591 Å, *c* = 27.644 Å.^51^, ^52^ The phase transition temperatures of both the cooling and heating cycle for all samples without and with Cl doping are summarized in Figure 3e. As the Cl ratio increases, the phase transition temperature of the heating cycle first increases from 220 K for the I:Cl ratio of 1:0 to 250 K for the ratio of 1:3 and then decreases to 240 K for the ratio of 1:8 while the transition temperature of the cooling cycle continuously decreases from 205 K for the ratio of 1:0 to 195 K for the ratio of 1:8. As a result, the temperature span between the cooling and heating cycle also increases from 20 K for the I:Cl ratio of 1:0 to 45 K for the ratio of 1:3 and then slightly decreases to 40 K for the ratio of 1:8 (Figure 3f). The presence of the hysteresis indicates that a metastable state exists within a temperature range and the crystal structure is switchable within this temperature range. The sudden shift of the emission peak near the phase transition temperature and the presence of the huge hysteresis between the cooling and heating cycle unambiguously indicate that the orthorhombic‐to‐orthorhombic phase transition observed here is a first‐order solid–solid phase transition.^53^

To further confirm the Cl incorporation induced the change of the phase transition temperature, DSC measurement has been carried out, which is a direct technique to probe the phase transition. It should be noted that we only show the data for the heating cycle since the phase transition temperature for our samples during the cooling cycle is out of the temperature range of the DSC measurement here. The DSC plot also reveals that the phase transition indeed takes place and the transition temperature first increases from 268 K for the I:Cl ratio of 1:0 to 270 K for the ratio of 1:3 and then slightly decreases to 269 K for the ratio of 1:8, the trend of which agrees with that of PL emission peak for the heating cycle. Nevertheless, the measured phase transition temperatures for all samples by DSC are quite different from that estimated from PL spectra, which might be due to the size‐dependent phase transition temperature as previously reported.^54^ While the bulk samples were used for DSC measurement, PL studies were carried out on the exfoliated microplates to avoid interference effect (Figure S7, Supporting Information). Overall, the DSC studies further confirm that the phase transition is present, and the transition temperature can be tuned via Cl incorporation, agreeing well with the results obtained by temperature‐dependent PL measurements.

One possible explanation for such tunable phase transition by Cl incorporation is that the Cl incorporation alters the crystal lattice constant and/or distorts the inorganic octahedrons, which would further affect the conformational changes within the ammonium chains or order–disorder transitions of the ammonium chains along their longitudinal axis during the process of phase transition (Figure S8, Supporting Information).^48^ As a result, both the transition temperature and transition temperature span between the cooling and heating cycle are tuned by the Cl doping. Nevertheless, the underlying mechanism remains elusive and further investigations are demanding.

Finally, we have studied how the Cl incorporation influences the charge transport of the as‐synthesized materials, which are indispensable for improving the performance of the optoelectronic devices and designing new device structures.^55^ We fabricated back‐gated field‐effect transistor (FET) devices of (BA)_2_SnI_4−_ *_x_*Cl*_x_* microplates on a 300 nm SiO_2_/Si substrate with a channel length of 20 µm and SiO_2_ layer working as dielectric material. **Figure** **4**a displays the schematic of a bottom‐gate, bottom‐contact device with a thin layer of mechanically exfoliated (BA)_2_SnI_4−_ *_x_*Cl*_x_* microplates serving as the channel semiconductor. The detailed device fabrication process can be found in the Experimental Section. The inset of Figure 4b exhibits an optical image of a type (BA)_2_SnI_4−_ *_x_*Cl*_x_* microplate FET device with an I:Cl of 1:8. Unlike the films‐based perovskite FET, in which the presence of grain boundaries and other defects can severely hinder to extract the intrinsic transport parameters, the exfoliated 2D perovskite microplates are single crystals with less defects and thus beneficial for investigating their intrinsic electronic properties.^56^ Similar to the PL measurements, the as‐fabricated devices were immediately put into the vacuum chamber to minimize the sample degradation. All electrical measurements were carried out in dark from 77 K to room temperature. For each sample with different I:Cl ratio, at least six devices have been measured.

**Figure 4:**
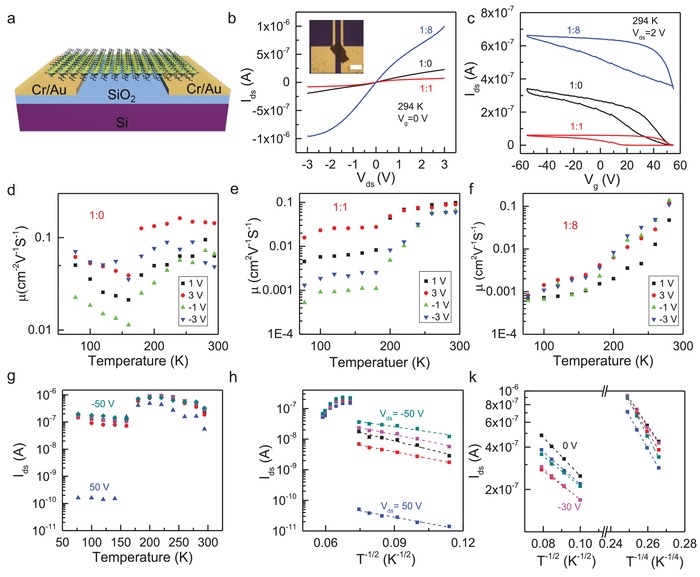
The charge transport properties of the (BA)_2_SnI_4−_ *_x_*Cl*_x_* microplates: a) Schematic of the bottom‐gate, bottom‐contact 2D perovskite microplate field‐effect transistor fabricated on a 300 nm SiO_2_/Si substrate with 5 nm Cr/50 nm Au as contact. b,c) The output and transfer characteristics of a field‐effect transistor based on (BA)_2_SnI_4−_ *_x_*Cl*_x_* crystal microplate with the I:Cl ratios of 1:0, 1:1, and 1:8 at room temperature. The inset of (b) shows an optical image of a typical device. The channel length is around 20 µm and the scale bar is 40 µm. d–f) The temperature‐dependent field‐effect hole mobility *µ* for (BA)_2_SnI_4−_ *_x_*Cl*_x_* crystals with various Cl incorporation concentration under different source–drain bias. g–k) The temperature‐dependent source–drain current under different applied gate voltages for (BA)_2_SnI_4−_ *_x_*Cl*_x_* crystals with various Cl incorporation concentration of 1:0 (g), 1:1 (h) and 1:8 (k).

Figure 4b shows the output characteristics (*I*_ds_ vs *V*_ds_) of the 2D perovskite microplate devices with various Cl incorporation concentration at room temperature without applying a gate voltage. To compare among devices, we have normalized the source–drain current by the cross‐section area of the respective microplate. The nonlinearity of the *IV* curves indicates that the electrical contact is not optimal. Importantly, the source–drain current first decreases and then continuously increases with the increasing Cl incorporation concentration, suggesting that the Cl doping could improve the conductivity of (BA)_2_SnI_4−_ *_x_*Cl*_x_* crystals for a higher Cl incorporation concentration which would be beneficial for the device applications (Figure S9, Supporting Information). For all devices, the source–drain current *I*_ds_ continuously increases with increasing negative gate voltage and decreases with increasing positive gate voltage, indicating a p‐type semiconductor behavior.

Figure 4c shows the transfer characteristics of the exfoliated (BA)_2_SnI_4−_ *_x_*Cl*_x_* microplate devices with various Cl incorporation concentration at a source–drain bias of 2 V at room temperature. All devices exhibit p‐type semiconductor behavior.^57^ The trend of the current change with the Cl incorporation concentration is the same as that of the source–drain current in Figure 4b. Nevertheless, there is no gate response for the devices with the I:Cl ratio of 1:3 and 1:6 (Figure S10, Supporting Information). A large hysteresis is observed for all devices (Figure 4c). Unlike 3D perovskites where the huge hysteresis can be ascribed to the ion migration, previous studies have proven that the ion migration has been greatly suppressed in both in‐plane and out‐of‐plane in 2D layered perovskites.^58^ Thus, it is unlikely that ion migration could contribute to the hysteresis observed here and the hysteresis in our devices is more likely due to the trapping/detrapping of charge carriers at the interfaces.^59^, ^60^

The field‐effect mobility *µ* was extracted from the positive sweeping of the transfer characteristics using the following formula: $\mu =\frac{L}{WCV_{\mathrm{ds}}}\frac{dI_{\mathrm{ds}}}{dV_{g}}$, where *L* is the channel length, *W* is the channel width, *C* is the gate‐channel capacitance, *V*_ds_ is the source–drain voltage, *I*_ds_ is the source–drain current, and *V*_g_ is the gate voltage.^61^ Figure 4d–f displays the temperature‐dependent field‐effect hole mobility for the exfoliated (BA)_2_SnI_4−_ *_x_*Cl*_x_* microplate devices with various Cl incorporation concentration under different source–drain bias. Without Cl incorporation (Figure 4d), the field‐effect hole mobility continuously decreases about one order with the increasing temperature from 77 to 160 K, and then shows a sudden jump around 180 K. Afterward, the field‐effect hole mobility starts to increase again with further increasing temperature. The sudden jump in mobility is due to the orthorhombic‐to‐orthorhombic phase transition as discussed above. While the decreases of mobility below 160 K with increasing temperature can be attributed to the reduced carrier–phonon scattering, the increases of the mobility with temperature above 180 K are probably owning to the in‐gap state induced thermal activated charge transport behavior or the formation of small polarons resulting from the strong electron–phonon interaction.^62^

In contrast, the field‐effect hole mobility continuously increases with increasing temperature for Cl incorporated samples (Figure 4e,f). For the samples with the I:Cl ratio of 1:1, the mobility first gradually increases with temperature between 77 and 180 K, then shows a sudden jump around 200 K and starts to increase again with a larger slope, and finally saturates above 260 K. The mobility of the samples with the I:Cl ratio of 1:8 exhibits the similar behavior but only has two slope and a weak jump around 200 K. While the jump of mobility can be ascribed to the phase transition observed in the temperature‐dependent PL studies discussed above, the continuous increase of the mobility might be originated from the in‐gap state induced thermal activated charge transport behavior. Similar transport behavior has been observed in the exfoliated MoS_2_, organic semiconductors, or amorphous silicon,^63^ which was attributed to the presence of disorders that introduce the extra charge impurity scattering and thus the thermal activated charge transport behavior.^64^ In our cases, we believe the incorporation of Cl would introduce the impurities and in‐gap states as well as induce the lattice deformation changing the local electronic structures and thereby leads to the thermal activated transport behavior. The slope of the mobility versus temperature for samples with different I:Cl ratios might be related to the degree of the lattice deformation, and the density of impurities and in‐gap states induced by Cl incorporation.

Furthermore, we also extracted the temperature‐dependent source–drain current under different applied gate voltages to confirm the proposed charge transport mechanism above. A clear sharp change of the source–drain current was observed for all three different sorts of samples, which is due to the phase transition (Figure 4g–i). It should be noted the observed phase transition temperatures extracted from charge transport studies are much lower than those from temperature‐dependent PL studies, which might arise from the much thinner microplates we used to fabricate the electronic devices. The size‐dependent phase transition temperature has been demonstrated and the thinner sample gives a lower transition temperature.^61^ Since it is difficult to transfer very thick microplates by using aligned and transferred technique, the thickness of the microplates we used is below 100 nm (Figure S11, Supporting Information), resulting in a lower phase transition temperature.

For microplates without Cl incorporation, the source–drain current continuously decreases with the increasing temperature for the temperature ranges both after and before phase transition, which originates from the enhanced carrier–phonon interaction with the increase of the temperature (Figure 4g).^65^ The similar trend was observed above 200 K before phase transition for the microplates with the I:Cl ratio of 1:1 for the same reason; however, after phase transition ln(*I*_ds_) is linear in *T* ^−1/2^ from 77 to 180 K (Figure 4h). For the devices with the I:Cl ratio of 1:8, ln(*I*_ds_) is linear in *T* ^−1/2^ from 77 to 160 K and linear in *T* ^−1/4^ from 200 to 294 K. While the *T* ^−1/4^ dependence behavior above 200 K before phase transition for the devices with the I:Cl ratio of 1:8 might be identified as Mott‐VRH (Mott‐variable range hopping) where transport occurs by thermally assisted hopping between localized electronic states similar to the transport in amorphous Ge, Si^63^ and 2D materials such as disordered graphene nanoribbons, MoS_2_, and FeS_2_.^66^, ^67^, ^68^ The *T* ^−1/2^ dependence behavior below 200 K for samples with the I:Cl ratios of both 1:1 and 1:8, however, could be interpreted in a number of ways, particularly conventional Efros–Shklovskii (ES) VRH.^69^ Those primary results clearly indicate that the charge transport behavior can be readily tuned by adjusting the Cl incorporation concentration.^70^ Nevertheless, further studies are needed to unveil the underlying transport mechanism in detail.

In conclusion, we have synthesized the lead‐free Sn‐based 2D perovskite (BA)_2_SnI_4−_ *_x_*Cl*_x_* with various Cl incorporation concentration. Cl element has been successfully incorporated into the crystal lattice of (BA)_2_SnI_4_, which is the first time to dope Cl into the crystal lattice of the perovskite materials by solution method. It is further demonstrated that the Cl incorporation concentration would alter the morphology of the as‐synthesized crystals, emission peaks, phase transition behavior, and finally charge transport behavior due to the Cl incorporation induced lattice distortion, doping effect, and impurities. Our findings offer an efficient strategy to incorporate the Cl into the lead‐free Sn‐based 2D perovskites with a desired Cl incorporation concentration to improve their performance of electronics and optoelectronics by low‐cost solution method. In addition, this controllable tunability of the Cl incorporation concentration provides not only an extra degree of freedom to modify the properties of the Sn‐based lead‐free 2D perovskites for optotelectronic applications, but also sheds light on the preparation of Sn‐based 2D perovskite thin films with Cl incorporation for the large‐scale solar cells and light‐emitting devices with improved performance.

## Experimental Section

*Material Preparation*: The Sn‐based 2D perovskite bulk crystals with various Cl incorporation concentration were synthesized by using the method similar to previous report except that HI solution was replaced by an excess of aqueous HCl/H_3_PO_2_ solution in order to incorporate the Cl element into the (BA)_2_SnI_4_ crystals. In details, SnO powder (202 mg, 1.5 mmol) was dissolved in a mixture of 57% w/w aqueous HI solution (3.5 mL) and 50% aqueous H_3_PO_2_ (0.75 mL) by heating to 150 °C under constant magnetic stirring for about 5 min, forming a bright yellow solution. Subsequently, *n*‐CH_3_CH_2_CH_2_CH_2_NH_3_ (1.5 mL, 4 mmol) in 57% w/w aqueous HI solution was then added into the resultant solution and held for another 10 min. The stirring was then discontinued, and the solution was left to cool to room temperature naturally. The as‐grown crystals were finally isolated by suction filtration and thoroughly dried at 60 °C.

For the synthesis of the Cl incorporated samples, the exact same procedure was adopted except that 57% w/w aqueous HI solution (3.5 mL) was replaced by 37% w/w aqueous HCl solution (1.5 mL) and BAI solution (1.5 mL, 4 mmol) for I:Cl = 1:1; HCl (3 mL) and BAI (1 mL) for I:Cl = 1:3; HCl (3 mL) and BAI (0.5 mL) for I:Cl = 1:6; and HCl (4 mL) and BAI (0.5 mL) for I:Cl = 1:8.

*Material Characterizations*: Powder X‐ray diffraction measurements were recorded using a Bruker D2 PHASER (Cu Kα, λ = 0.15419 nm, nickel filter, 25 kV, 40 mA). Optical microscopy images were collected by an Olympus BX53M system microscope. The SEM images were acquired on a JEOL 7001F field emission scanning electron microscope equipped with energy‐dispersive X‐ray spectrometer (EDS). DSC measurement was carried out by a PSTA449F3, Netzsch, Germany. XPS was recorded on a Shimadzu/Kratos Axis‐Ultra DLD‐600W instrument equipped with Al/Mg Kα radiation. The photoluminescence measurements were performed in backscattering configuration using a Horiba HR550 system equipped with a 600 g mm^−1^ excited by a 473 nm solid‐state laser with a power of 0.2 µW. The temperature‐dependent photoluminescence measurements were carried out in a liquid nitrogen continuous flow cryostat (Cryo Industry of America) coupled with a temperature controller. The reflection measurement was carried out in a Horiba HR550 system with a 600 g mm^−1^ grating illuminated by a 100 W halogen tungsten lamp.

*Device Fabrication and Electrical Measurement*: 5 nm Cr/50 nm Au two‐probe electrodes were defined on a 300 nm SiO_2_/Si wafer by photolithography and followed by electron beam evaporation and lift‐off process. The 2D perovskite microplates were mechanically exfoliated from their respective bulk crystals by using Scotch tape. The exfoliated 2D perovskite microplates were subsequently transferred onto the predefined electrodes under the aid of microscopy as previously reported.^71^ The as‐prepared devices were immediately put into the vacuum chamber for further temperature‐dependent electrical measurements, which was carried out in a probe station (Lakeshore, PS100) coupled with a precision source/measurement unit (Agilent B2902A).

## Conflict of Interest

The authors declare no conflict of interest.

## Supporting information

Supplementary
