# Supplementary material for: The Role of Chloride Incorporation in Lead‐Free 2D Perovskite (BA)2SnI4: Morphology, Photoluminescence, Phase Transition, and Charge Transport
Source: Adv Sci (Weinh). 2019 Jan 20;6(5):1802019. doi: 10.1002/advs.201802019 (PMC6402407; doi:10.1002/advs.201802019)
Supplement: Supplementary file 1 — Supplementary [file ADVS-6-1802019-s001.pdf]

Copyright WILEY-VCH Verlag GmbH & Co. KGaA, 69469 Weinheim, Germany, 2019.

ADVANCED SCIENCE
Open Access

Supporting Information

for *Adv. Sci.,* DOI: 10.1002/advs.201802019

The Role of Chloride Incorporation in Lead-Free 2D Perovskite $(BA)_2SnI_4$: Morphology, Photoluminescence, Phase Transition, and Charge Transport

*Jun Wang, Hongzhi Shen, Waicai Li, Shuai Wang, Junze Li, and Dehui Li**

Copyright WILEY-VCH Verlag GmbH & Co. KGaA, 69469 Weinheim, Germany, 2019.

Supporting Information

The role of chloride incorporation in lead-free 2D perovskite $(BA)_2SnI_4$: morphology, photoluminescence, phase transition and charge transport.

Jun Wang, Hongzhi Shen, Waicai Li, Shuai Wang, Junze Li and Dehui Li*

Dr. Jun Wang, Hongzhi Shen, Waicai Li, Shuai Wang, and Junze Li
School of Optical and Electronic Information, Huazhong University of Science and Technology, Wuhan, 430074, China
Prof. Dehui Li
School of Optical and Electronic Information and Wuhan National Laboratory for Optoelectronics, Huazhong University of Science and Technology, Wuhan, 430074, China
E-mail: ~~dehuili@hust.edu.cn~~.

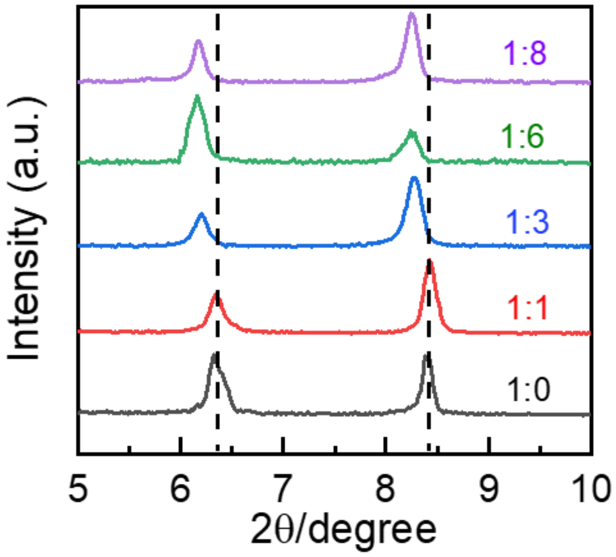

Figure S1. The magnified low angle XRD patterns for samples with different I/Cl ratios

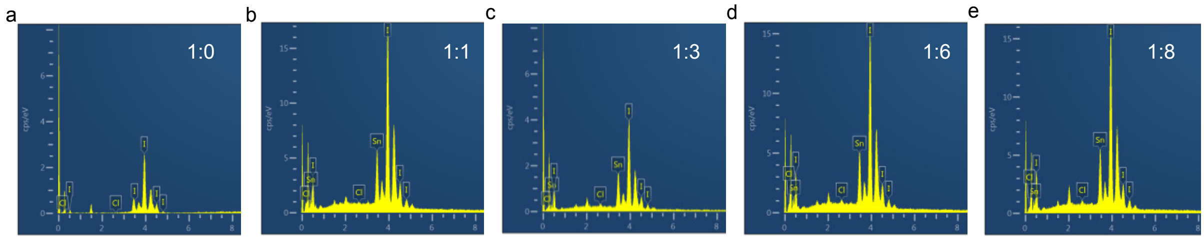

I:Cl	1:1	1:3	1:6	1:8
At%	0.29	0.41	0.43	0.54

Figure S2. Energy-dispersive X-ray spectroscopy (EDS) analysis of $(BA)_2SnI_{4-x}Cl_x$ with different I/Cl ratios. The table shows the atomic percentage of the Cl element, which gradually increases as the ratio of I to Cl increases in precursor solution.

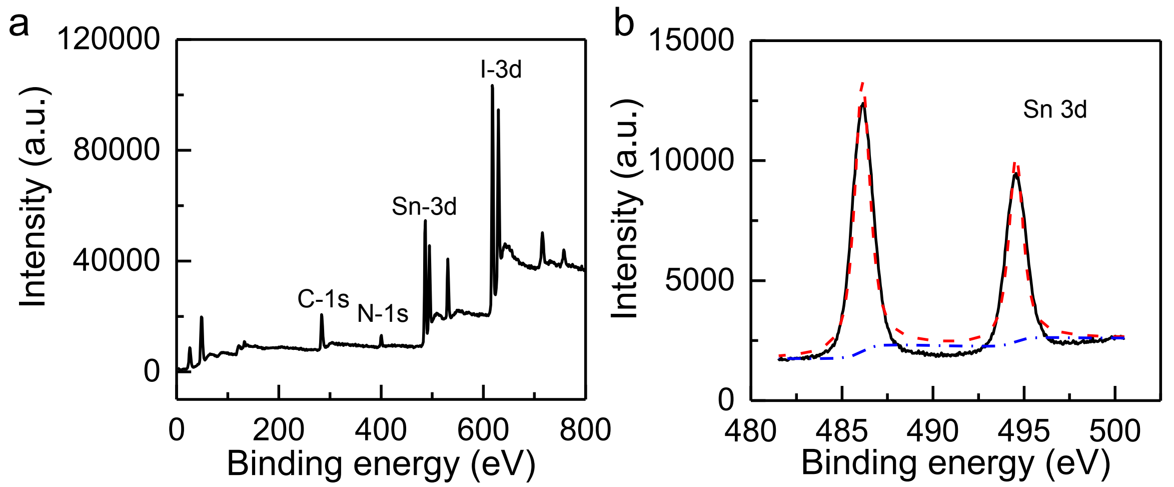

Figure S3. a) XPS spectrum of pure $(BA)_2SnI_4$ without Cl incorporation. b) Zoom-in XPS spectrum of Sn (3d) peak shows the absence of Sn^{4+}.

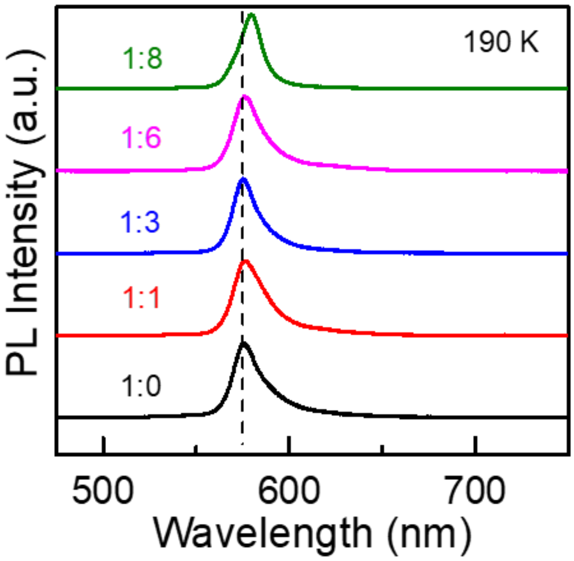

Figure S4. The steady-state PL of as-synthesized $(BA)_2SnI_{4-x}Cl_x$ crystals at 190 K when the phase transition has been completed.

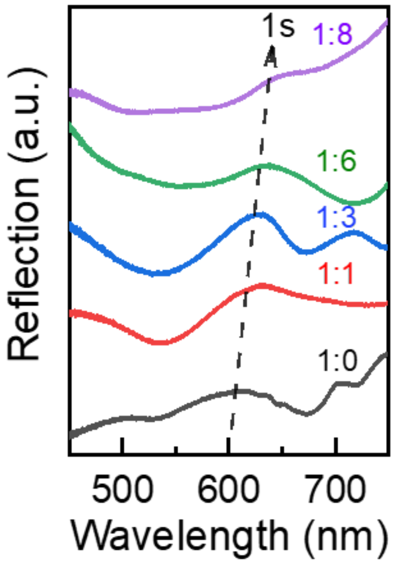

Figure S5. Reflection spectra of the as-synthesized samples with different I/Cl ratios.

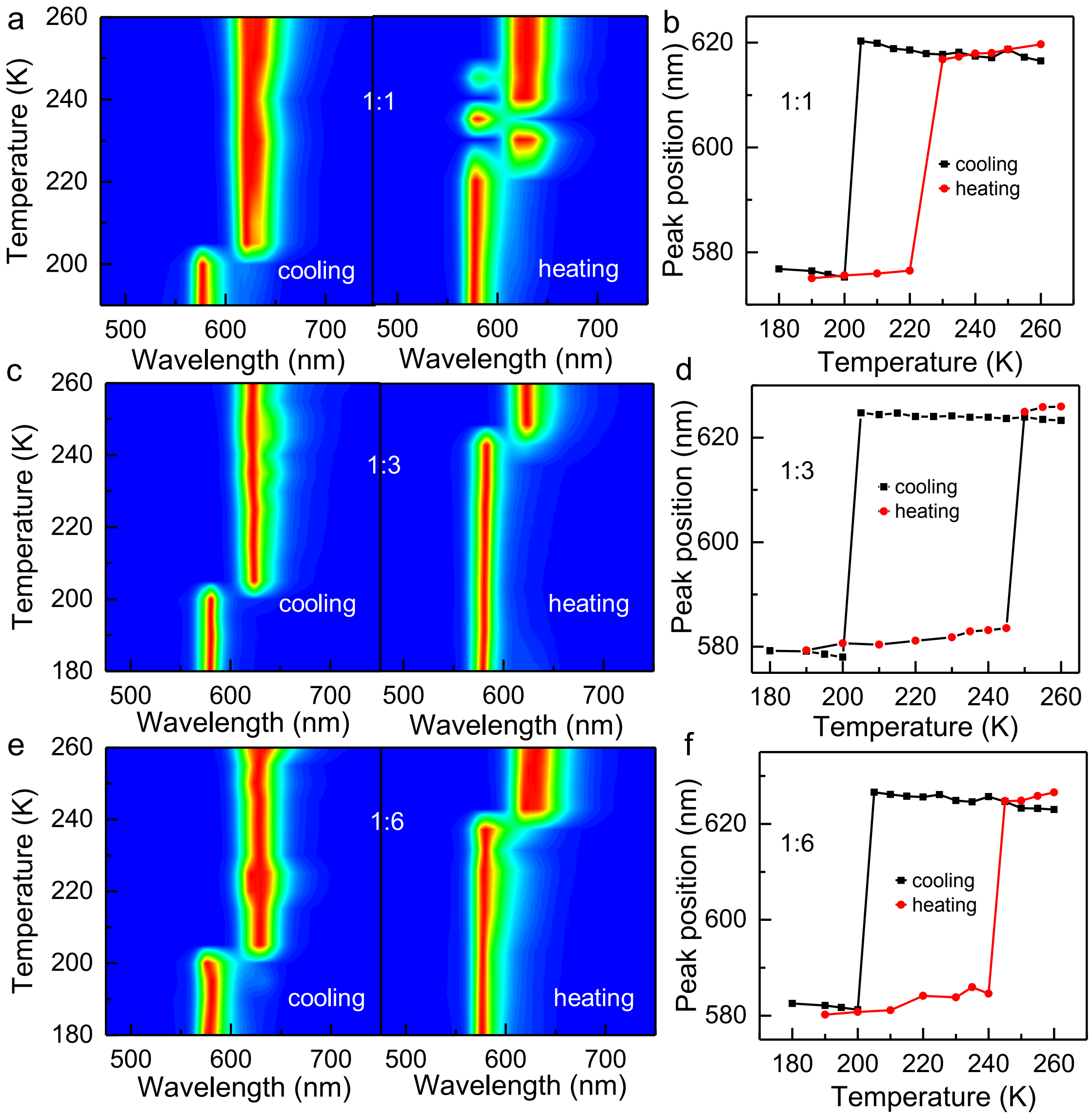

Figure S6. Temperature-dependent photoluminescence map of $BA_2SnI_{4-x}I_x$ crystals for the cooling and heating cycle for the $(BA)_2SnI_{4-x}Cl_x$ crystals with the I/Cl ratio of 1:1 a), 1:3 c) and 1:6 e). The temperature dependent emission peak of $(BA)_2SnI_{4-x}Cl_x$ crystals with the I/Cl ratio of 1:1 b), 1:3 d) and 1:6 f) extracted from a, c, e).

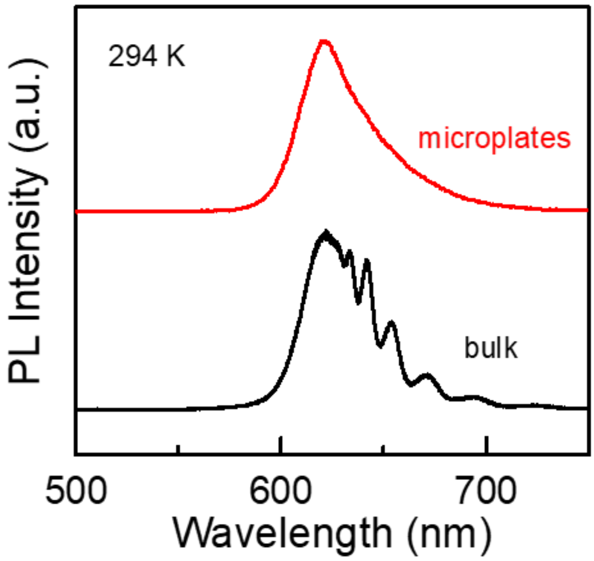

Figure S7. The steady-state PL spectra of $(BA)_2SnI_{4-x}Cl_x$ bulk and exfoliated microplates with the I/Cl ratio of 1:1 at room temperature.

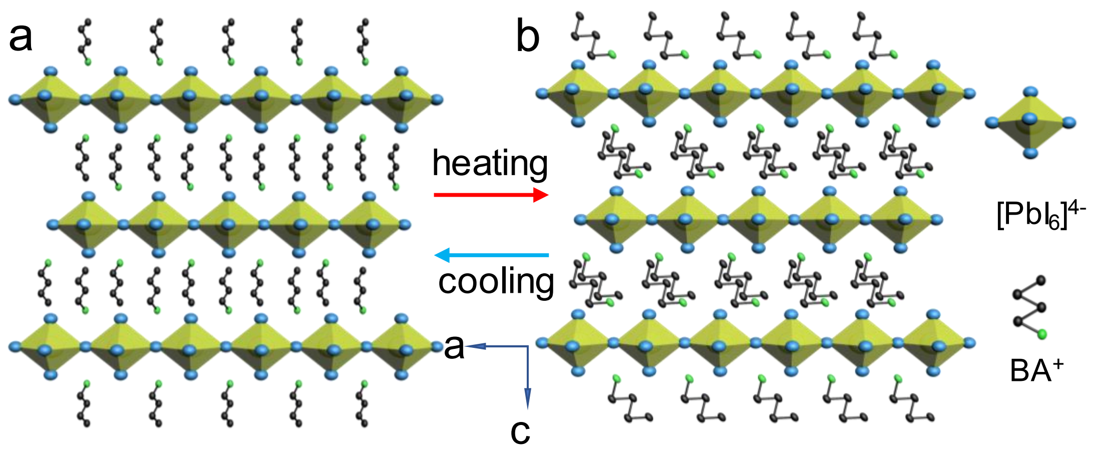

Figure S8. Molecular structures viewed along the b axis at a) 294 K and b) 77 K.

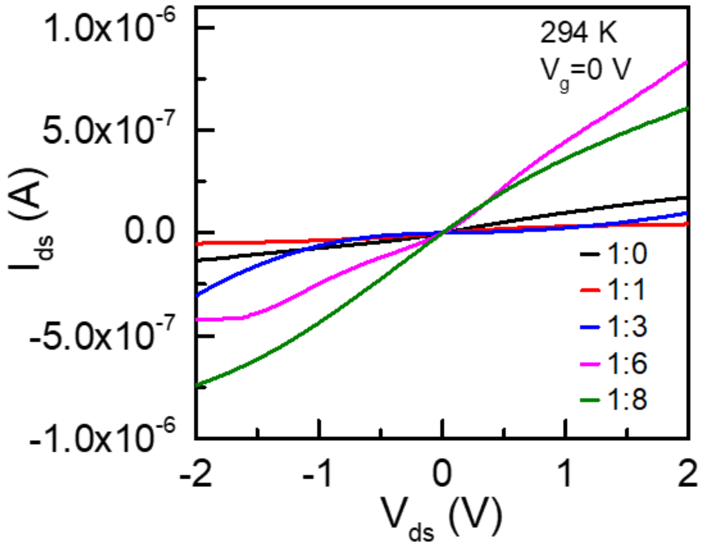

Figure S9. The source-drain current I_{ds} versus V_{ds} at 294 K for the as-synthesized $(BA)_2SnI_{4-x}Cl_x$ crystals with different I/Cl ratio.

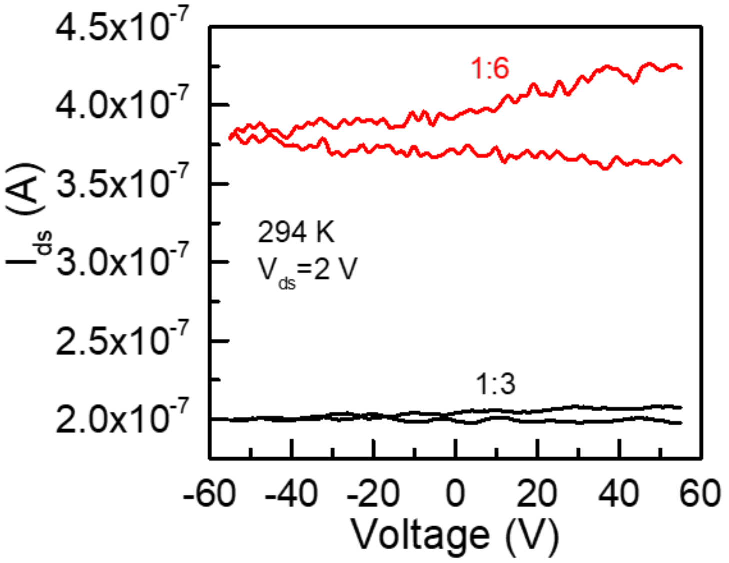

Figure S10. Transfer characteristics of the field-effect transistors based on $(BA)_2SnI_{4-x}Cl_x$ crystal microplates with the I/Cl ratio of 1:6 and 1:8 at room temperature. There is no gate response for these two ratios.

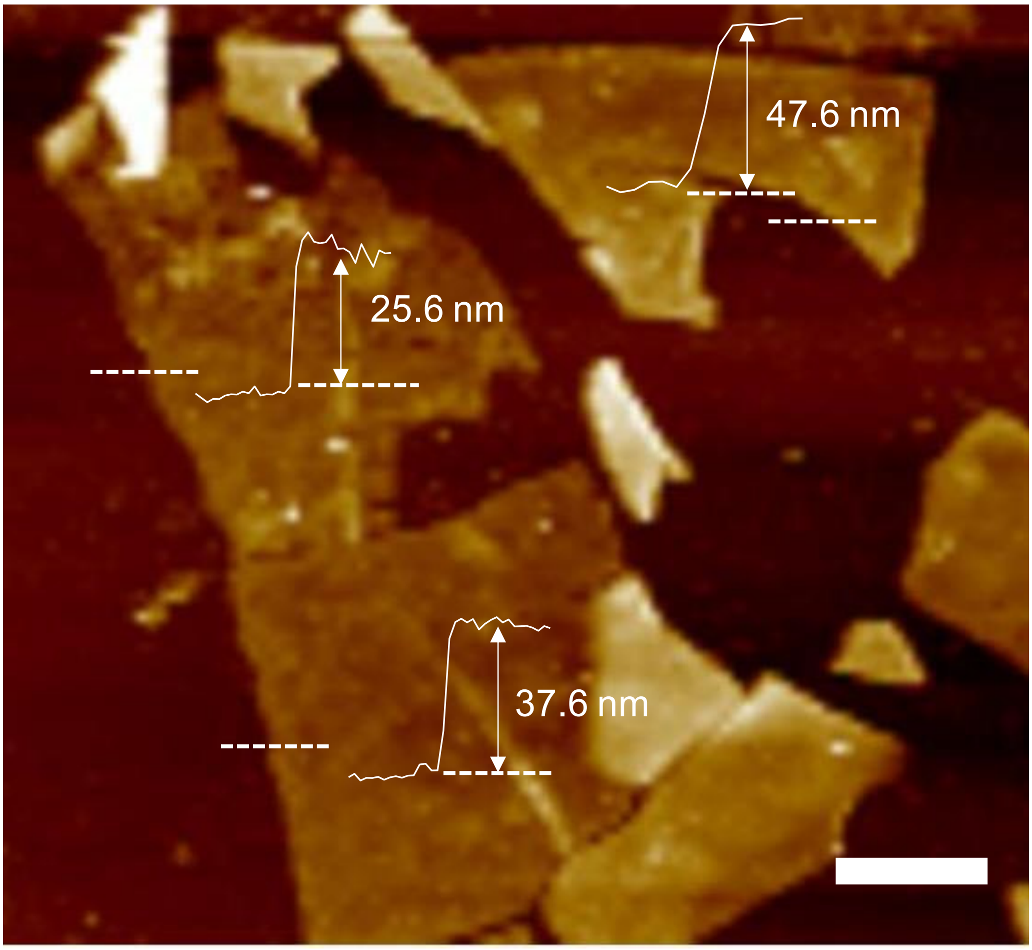

Figure S11. AFM images of the exfoliated $(BA)_2SnI_{4-x}Cl_x$ microplates with the I/Cl ratio of 1:3 for the back-gated FET devices. The scale bar is 20 μm.
